# DelicacyNet for nutritional evaluation of recipes

**DOI:** 10.3389/fnut.2023.1247631

**Published:** 2023-09-14

**Authors:** Ruijie Li, Peihan Ji, Qing Kong

**Affiliations:** ^1^College of Computer Science and Technology, Faculty of Information Science and Engineering, Ocean University of China, Qingdao, Shandong, China; ^2^Haide College, Ocean University of China, Qingdao, Shandong, China; ^3^College of Food Science and Engineering, Ocean University of China, Qingdao, Shandong, China

**Keywords:** recipe, nutrition, computer vision, transformer, environment features

## Abstract

In this paper, we are interested in how computers can be used to better serve us humans, such as helping humans control their nutrient intake, with higher level shortcuts. Specifically, the neural network model was used to help humans identify and analyze the content and proportion of nutrients in daily food intake, so as to help humans autonomously choose and reasonably match diets. In this study, we formed the program we wanted to obtain by establishing four modules, in which the imagination module sampled the environment, then relied on the encoder to extract the implicit features of the image, and finally relied on the decoder to obtain the required feature vector from the implicit features, and converted it into the battalion formation table information through the semantic output module. Finally, the model achieved extremely high accuracy on recipe1M+ and food2K datasets.

## 1. Introduction

Food is the priority of the people. The six nutrients contained in food not only constitute the body, but also produce energy to maintain human growth after metabolism (1, 2). Every day, billions of people share comments related to food on social networks (3), which shows that food has irreplaceable value in our life. There has been a shift in dietary guidelines around the world from focusing on single nutrients to focusing on food patterns, food groups and dietary components, which provides an opportunity for our research (4–6). Different cooking techniques and cooking time will produce different kinds of food. Even though there are obvious differences between Chinese and Western food cultures, the data of these images were included in our training database (Food2K, Recipe1M+, and USDA National Nutrient Database), covering all kinds of food to the greatest extent.

Food image recognition is a basic task of food computing (7). By introducing large-scale Food databases, such as Food2K dataset, Recipe1M+ dataset, and ETH Food-101, Vireo Food-172 (8) and ISIA Food-500 datasets (9), it becomes a branch of fine-grained visual recognition (10). Recipe1M+ is the largest publicly available recipe dataset, which includes 13 million food images and more than 1 million cooking recipes, providing an opportunity to design high-capacity models on unified multimodal data (11, 12). The Food2K dataset involves 12 superclasses (such as vegetables, meat, barbecue, and fried foods, etc.) and 26 subcategories, containing both Eastern and Western dishes, increasing the number of images in the dataset by orders of magnitude. This dataset covers a wide range of diverse visual appearances, and pays attention to the global and local features of images. Therefore, the deep progressive mode of food recognition should be adopted, and the richer global background was integrated into the local background features to improve the retrieval accuracy (13).

With the development of interactive technology integrated with various food environments, in the field of human-computer interaction (HCI), people continue to challenge digital food technology, in order to improve food living standards more efficiently (14, 15). In the early 2020s, Transformer was widely used as a deep learning model utilizing an attention mechanism to increase the training speed of the model for creating musical rhythms (16), occluded image recovery (17), mathematical operations, etc. (18, 19). In medical research, integrating neural distance and texture-aware transformers can help improve prognostic prediction of pancreatic cancer (20). Then Vision Transformers (ViTs) emerged and replaced ConvNets as the state-of-the-art image classification model. Among the many improvements to ViT, the Swin Transformer counts as a very successful one. Aiming at the problem that the CV task is generally multi-scale pictures and the image resolution is large, the local attention calculation module LSA is creatively proposed, that is, the self-attention is calculated only within the window. Compared with ViT, the performance is also greatly improved, which improves the practicability of Transformer. In 2020, FAIR introduced ConvNext, a pure convolutional neural network with non-vit performance, and in 2023, ConvNext-v2 (21, 22) combines MAE from NLP to achieve SOTA performance on fundamental vision tasks.

Deep learning has been widely used in various fields currently. The pseudo-siamese network can effectively correct underwater non-uniform illumination images, separating the non-uniform illumination layer from the ideally illuminated image (23), thus achieving the purpose of enhancing image details and improving image quality, and thus achieve the purpose of enhancing image details and improving image quality. A novel physically-aware two-stream underwater image enhancement network, PA-UIENet (24), reduces errors when facing various underwater scenes by simulating image degradation and learning features of various underwater scenes. The Complementary Learning with Content Noise in Pharmaceutical Images (CNCL) strategy leads in visual quality and quantitative metrics by leveraging features unique to medical images (25).

The training of such computer programs is affected by datasets and domain gaps, which is also a great challenge for us. What we created is a model consisting of Encoder and Decoder, by preprocessing the input image, and then using the autoattentional encoder to adjust the final output target. To achieve this, we designed an innovative neural network structure DelicacyNet, which employed innovative structures and components for efficient semantic processing of food images. For the training model we used Food2K, Recipe1M+, and USDA National Nutrient Database. The performance of the model was evaluated using a custom loss function to monitor the performance of the model during the training and validation phases.

In traditional image recognition, fine-grained image recognition captures local image features with the help of semantic models, but dishes in food images cannot be discriminated by defining local images (26). Moreover, it adds extra difficulty to our procedural work due to the different sizes of dishes in the received food images. In addition, a large number of ingredients may be mixed in the same food, each with a different nutrient composition, which adds to the difficulty of direct discriminatory analyzes. Food image recognition is uniquely composite and difficult to segment, which poses a great challenge to our final output of nutrient content percentage.

## 2. Methods

It is a challenging task to extract nutritional information of food only from images. Throughout the cooking process, food undergoes a variety of physical and chemical processing methods and is mixed with various flavors, which will affect the original size, shape and color of food ingredients to a certain extent (27–29). Meanwhile, due to the different cooking process and storage time of food ingredients, the final image of the same food ingredient will also lead to color deviation, which brings great challenges to the image recognition and annotation.

We believe that global information cognition and local feature analysis are equally important in this problem, so we decide to extract global information and local features at the same time. Supplementary Figure S1 shows the basic structure of our model. The whole model consists of four parts: environment feature extraction module (EFE), encoder, decoder and semantic output module. The input of the model is in the form of an image, and the output is in the form of a text representing the nutrients in the food and their content.

### 2.1. Environment feature extraction module

In the process of food image processing, a difficult problem is the influence of environmental information, because in practical applications, the image will contain a lot of environmental information, which will interfere with the subsequent judgment of semantic extraction module. In order to better adapt to the needs of food image processing, this paper proposes a lightweight environmental feature extraction module (EFE), which can preserve the core feature information of the image as much as possible under the condition of realizing environmental denoising.

#### 2.1.1. HSI image conversion layer

**Figure 1:**
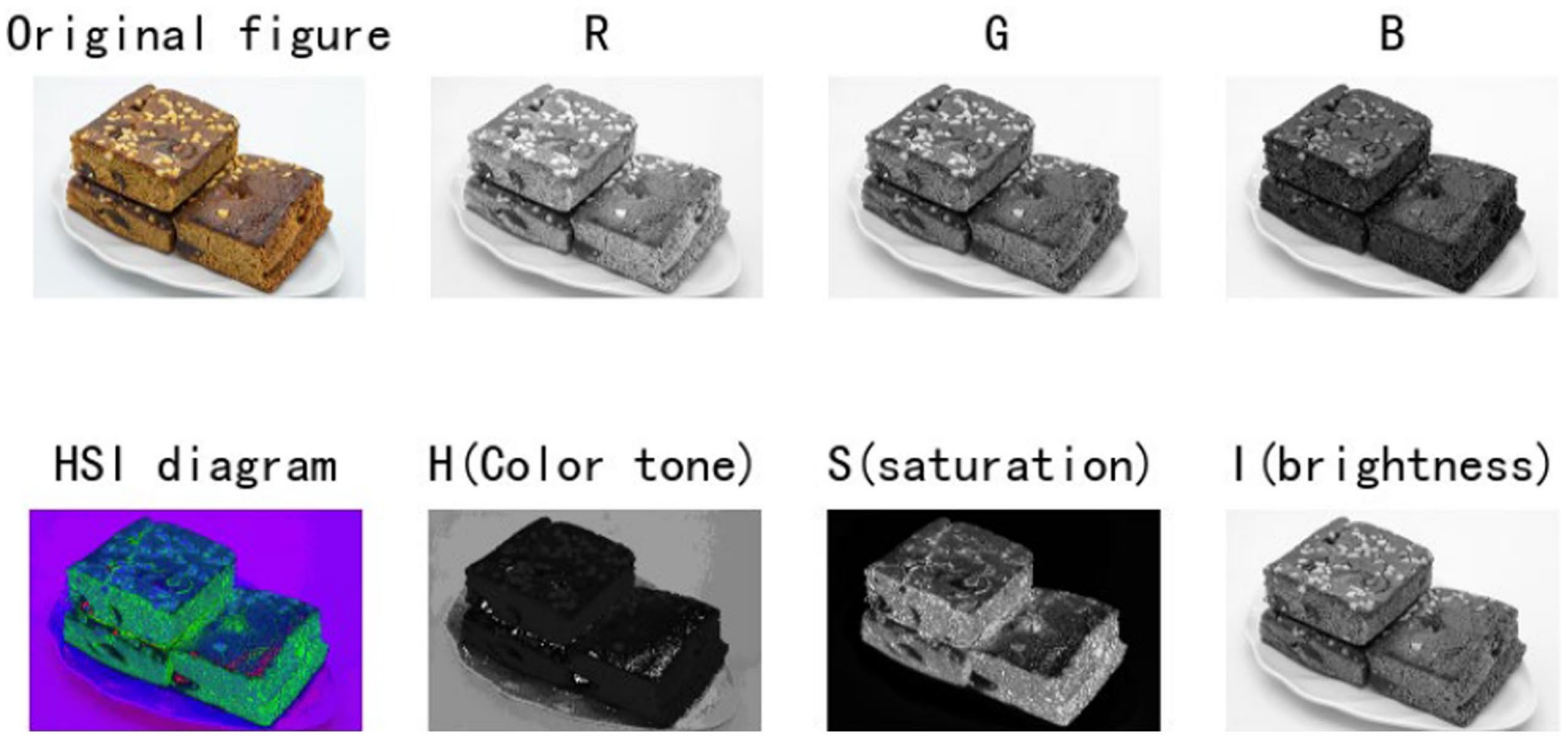
HSI image conversion layer.

In the study, we observe that the HSI image reconstructed by the formula has more information than the original RGB image (Figure 1), and it is also more conducive to the subsequent core feature extraction and background clutter removal. Therefore, at the beginning of the environment module, we added an HSI module to improve the performance of the model.

The image conversion formula is as follows:


$$r=\frac{R}{R+G+B},\;g=\frac{G}{R+G+B},\;b=\frac{B}{R+G+B}.$$

h=cos−10.5⋅r−g+r−br−g2+r−bg−b12h∈0,πforb≤g

h=2π−cos−10.5⋅r−g+r−br−g2+r−bg−b12h∈π,2πforb>g

$$s=1-3\cdot \min\;\left(r,g,b\right);s\in \left[0,1\right]$$

$$i=\left(R+G+B\right)/\left(3\cdot 255\right);i\in \left[0,1\right]$$

H=h×180/π;S=s×100andI=i×255

#### 2.1.2. Normalization layer MDN

**Figure 2:**
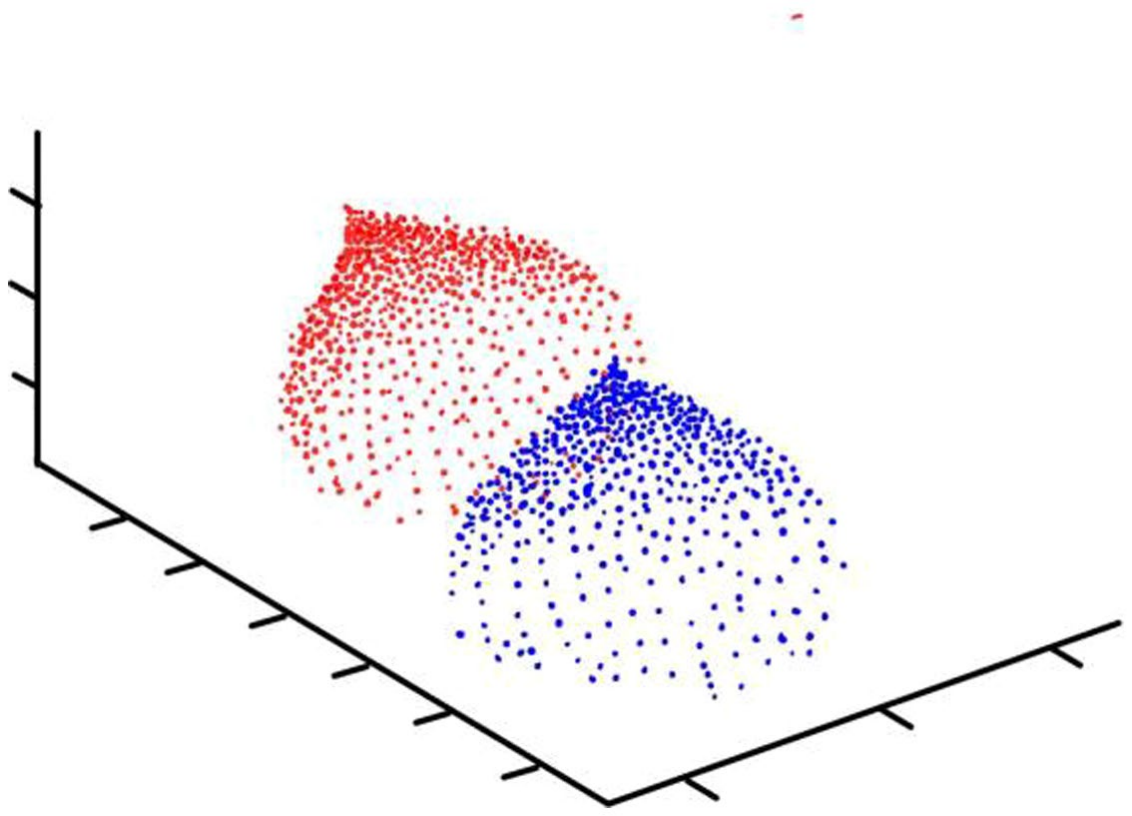
Mechanism of action of MDN.

MDN is a different kind of normalization layer from traditional thinking. It divides a number of intervals (or different distribution functions) based on the probability distribution of the information contained in the pixel (Figure 2). By dividing the interval, the visual subject of the image and the environment can be separated to further achieve good information extraction results.


$$KL\;\left(P||Q\right)=\sum P\left(x\right)\log\frac{P\left(x\right)}{Q\left(x\right)}$$

Specifically, we take advantage of the huge difference between the background and the visual center information, layer according to the convolution results of the convolution layer, and preprocess the function for each layer. The final normalization results in dividing each number by the norm of the plane of normal distribution into which it is partitioned. In detailed, it is:


$$norm\left(x\right)=\sqrt{x_{1}{}^{2}+x_{2}{}^{2}+\ldots +x_{n}{}^{2}}$$

$$l=norm\left(x^{\prime }\right)=\frac{\sqrt{x_{1}{}^{2}+x_{2}{}^{2}+\ldots +x_{n}{}^{2}}}{norm\left(x\right)}$$

$$=\sqrt{x_{1}\prime^{2}+x_{2}\prime^{2}+\ldots +x_{n}\prime^{2}}$$

#### 2.1.3. Environment feature extraction module

**Figure 3:**
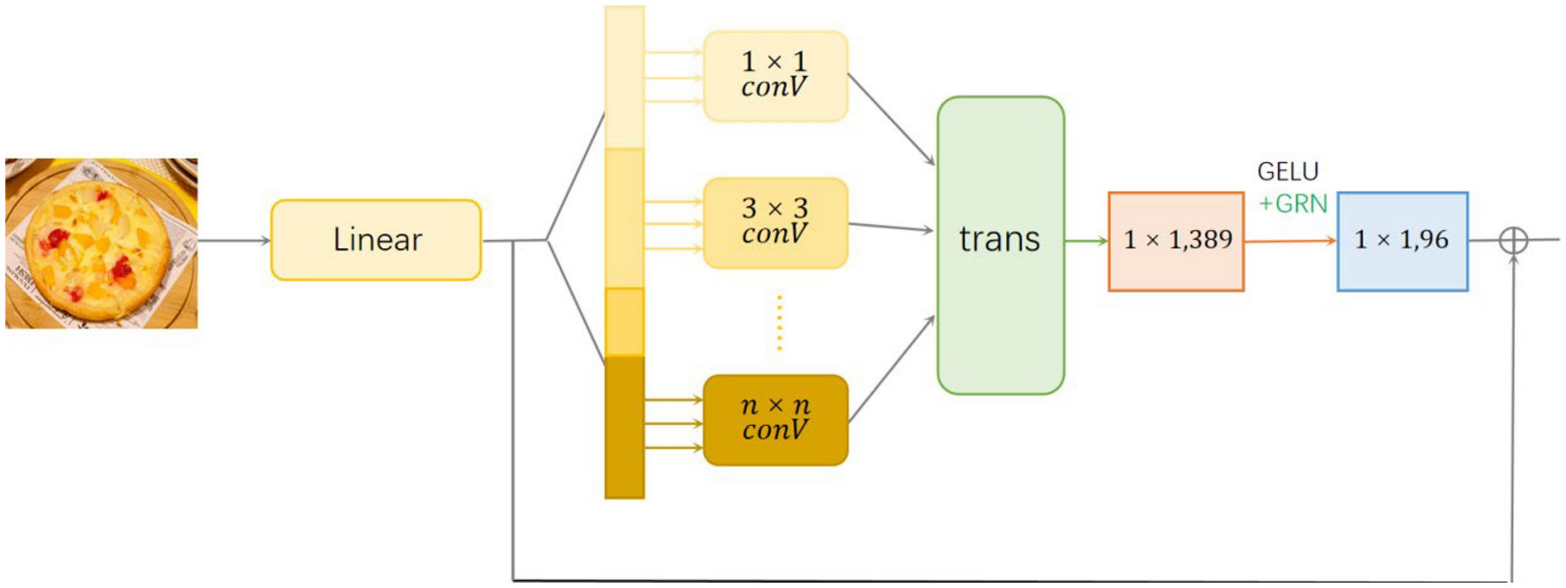
The environment feature extraction module.

We choose the fully convolutional neural network as the backbone network of the module. After mapping the image to one dimension through linear layers, in order to enhance the local sensibility and global difference judgment ability of the model, we introduce multiple convolution layers with different convolution kernel sizes, so that it can capture spatial features at multiple scales. Here, different convolution kernels are used to convolute the linear layer, respectively. After the convolution, tensors of different sizes and different numbers will be obtained according to the size of the convolution kernel. After recombining the tensors using the transformation layer, the 1 × 1 convolution kernel is used for convolution, and finally the 1 × 1 convolution kernel is used again after normalization (Figure 3).

### 2.2. Encoder

Only using the environment feature extraction module has a good image processing effect, but there is no way to associate the image with the semantic. The role of the encoder is to parse out the semantic information from the image. For better information extraction, we used multi-layer transformer architecture combined with Atrous Spatial Pyramid Pooling (ASPP) module (30). The ASPP module was able to capture multi-scale image features. In order to obtain richer local information, a residual structure was used between the encoder and the original image (31). The input of the encoder was the output feature tensor of the EFE module, and the output was the processed feature tensor (32).

The transformer module was refined into the following submodules:

*Linear projection layer*. The input feature matrix X was transformed into query (*Q*), key (*K*), and value (*V*) matrices, and global query ( $Q_{\mathrm{global}}$ ), global key ( $K_{global}$ ), and global value ( $V_{global}$ ) matrices (31).


$$Q=W_{q}*X$$

$$K=W_{k}*X$$

$$V=W_{v}*X$$

$$Q_{global}=W_{q_{global}}*X$$

$$K_{global}=W_{k_{global}}*X$$

$$V_{global}=W_{v_{global}}*X$$

*Focused attention module*. Based on the input *Q*, *K* and *V* matrices, the focused attention output C was calculated (33).


$$C=softmax\left(\frac{Q*K^{T}}{\sqrt{d_{k}}}\right)*V$$

*Independent attention module*. Based on the input $Q_{global}$ , $\;K_{global}$ and $V_{global}$ matrices, the independent attention output I was calculated (31).


$$I=softmax\left(\frac{Q_{global}*K_{global}{}^{T}}{\sqrt{d_{k}}}\right)*V_{global}$$

*MHSA module*. The focused attention output C and the independent attention output I were weighted summed and linearly transformed to obtain the multi-head self-attention output (34).


$$MHSA_{output}=W_{o}*\left(\alpha *C+\beta *I\right)$$

### 2.3. Decoder

The function of the decoder is to correspond the extracted implicit information features to the possible nutrients where these information features exist. We adopt a lightweight network with fully connected layer and residual layer as the main body to complete this structure. In order to simplify the calculation, the model uses a lightweight self-attention module Sup-HeadAttention. Based on the idea of deleting invalid branches in model compression, a supervision layer sup is added to each layer, which can detect inactive neurons in the process of dynamic training of the model. And it temporarily sleeps it with the current value for a certain number of epochs, thereby reducing the number of tokens that need to be passed to the next layer to achieve the purpose of speeding up inference. Compared with ViT, it has less computation and better performance.

**Figure 4:**
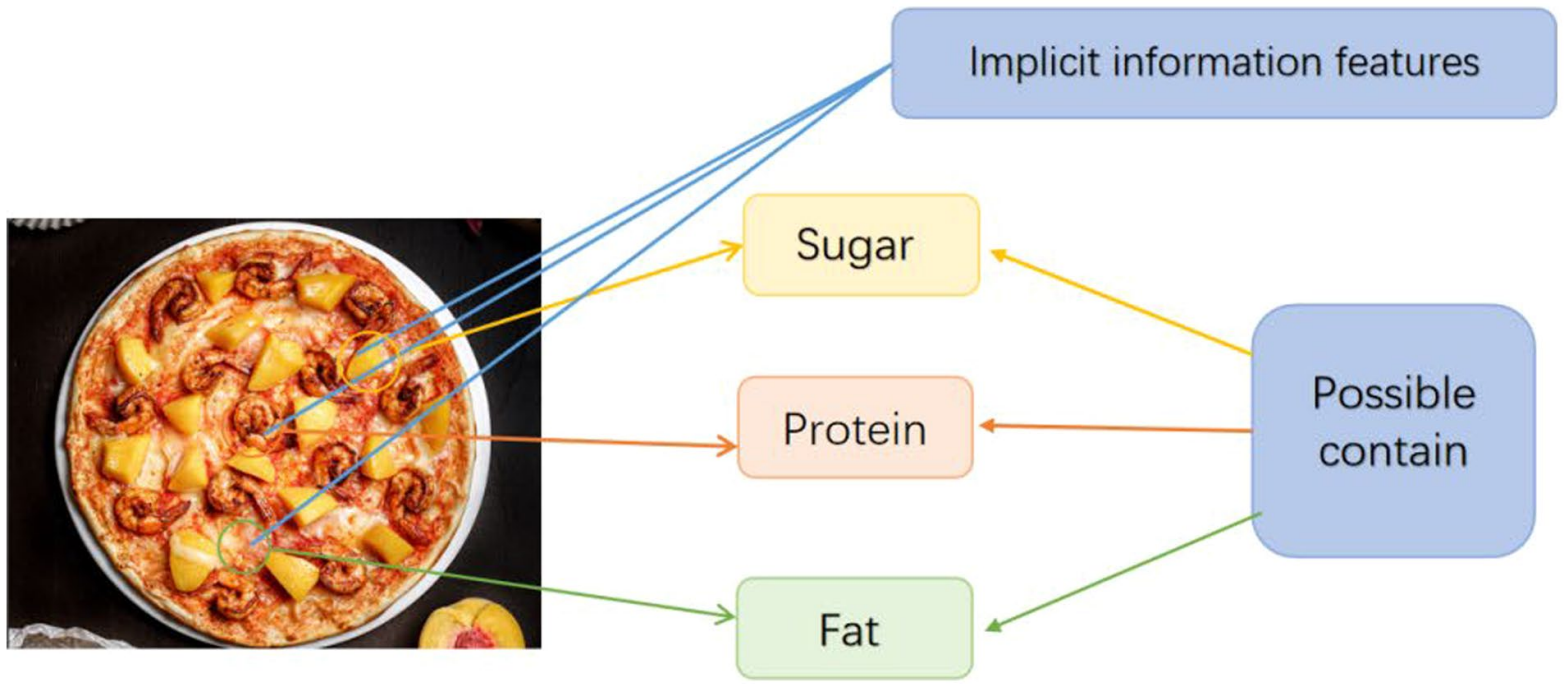
The function of decoder.

As shown in Figure 4, the model can extract a large number of implicit information features from the image, and the role of the decoder is to use these implicit information features to correspond to the possible content of the substance with the characteristic, and then determine the nutritional information in the image.

In the whole decoder structure, the model first passes through the self-attention module Sup-HeadAttention. Next, a dropout layer and a normalization layer are added to increase the generalization of the model. Finally, the output is passed to the fully connected layer to correspond to the corresponding nutrient information. The output of the decoder is a feature vector.

### 2.4. Semantic output module

This module was responsible for converting the output of the decoder into the predicted nutrient composition and content. To achieve this goal, we used multiple fully connected layers and Dropout layers to reduce overfitting. The last fully connected layer used a softmax activation function, which was used to predict the content of each nutrient. The input of the semantic output module was the output feature vector of the decoder, and the output was a numerical distribution representing the predicted nutrient content.

Code is available at https://github.com/nerakoo/DelicacyNet.

### 2.5. Experiments

#### 2.5.1. Implementation details

##### 2.5.1.1. Food2K data preprocessing

The images in the Food2K dataset may have different sizes. To feed them into our model, we need to resize all the images to a uniform size. Here we converted the image to the form (224, 224, 3). At the same time, we took normalization to map the image pixel values from the range [0, 255] to the range [0, 1], which helped the model converge and optimize better. In order to improve the generalization ability of the model, we used data augmentation techniques including random horizontal flip, random rotation, and random cropping to augment the training data. These operations were implemented using TensorFlow’s image processing functions.

##### 2.5.1.2. USDA national nutrient database data preprocessing

The specific information of USDA National Nutrient Database is that for each nutrient, and the ingredients contained in each food are given. In the actual network, we used the food2K database for image input. This caused the problem that the training set of the nutrient database and the training set of the image database were not named uniformly.

To integrate the USDA National Nutrient Database with the Food2K dataset, we need to create a mapping. Here, we used the mapping dictionary to convert the labels of the Food2K dataset to the food names corresponding to the USDA National Nutrient Database. The corresponding nutrient composition information was extracted from the USDA National Nutrient Database, and the food categories in the Food2K dataset were mapped to the foods in the USDA National Nutrient Database. In this way, after the input image, we predicted its nutritional content.

#### 2.5.2. Loss and activation functions

##### 2.5.2.1. Loss function

The Weighted Mean Absolute Error (WMAE) was used as the loss function. It took into account the importance of each nutrient and gives greater penalties to important nutrient prediction errors.

The WMAE loss function is defined as follows:


$$WMAE=\frac{1}{sum\left(w\right)}*\sum w_{i}*|y_{pred_{i}}-y_{true_{i}}|$$

Where sum(w) is the sum of the weight vectors w and is the absolute error between the predicted value and the true value of the ith nutrient.

By using the weighted mean absolute error loss function, we can better evaluate the performance of the model in predicting different nutrient contents, and adjust the weights to optimize the prediction ability of the model on key nutrients according to the actual needs.

##### 2.5.2.2. Activation function

GELU (Gaussian error linear unit) activation function was used in the model, which had better performance than ReLU (rectified linear unit), especially in fields such as natural language processing and computer vision. The derivative of ReLU at negative values is zero, which may lead to the vanishing gradient problem. In contrast, the derivative of GELU at negative values is not zero, which helps alleviate the vanishing gradient problem.

The mathematical expression of GELU is as follows:


$$GELU\left(x\right)=x*\Phi \left(x\right)$$

Here **Φ(x)** represents the cumulative probability distribution of the Gaussian distribution, that is, the definite integral of the Gaussian distribution over the interval **(−∞,x]**.

## 3. Result

### 3.1. Accuracy test

In the accuracy test of the model, we calculated and analyzed the nutrient contents of the foods in the pictures. Firstly, we estimated the content proportion of different categories of food in the picture, and looked up the content proportion of each type of nutrients in different types of food in the “Food safety – Food nutrient query table” (35), and further calculated the content proportion of each nutrient in the picture of the food. Then, we input the food picture into the model to get the results. Finally, we compared the estimated results as the true values with the model results to evaluate the accuracy of the model.

Table 1 shows that according to the input and output of the model, we can obtain the main nutritional composition and content information called Nutrient Reference Values (NRV) contained in the image by inputting it into the model. At the same time, we compared the output information with the calculated real information, and confirmed the accuracy and effectiveness of the model, which can meet the purpose of the original establishment of the model, that is, to help humans rationally mix diet.

**Table 1:** Comparison of the true results with the model prediction results.

Food image	Nutrient	NRV%	NRV% (DelicacyNet)
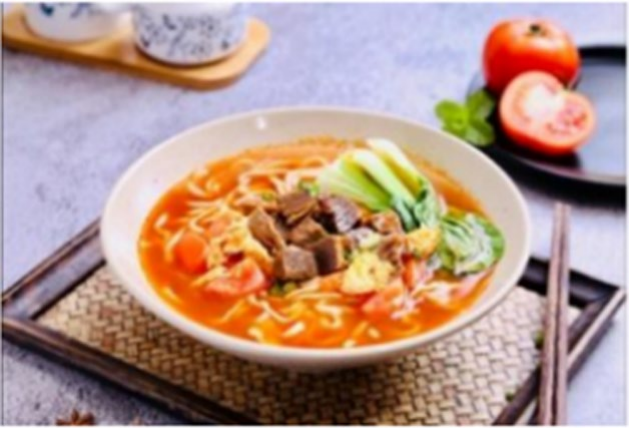	Protein	7.280	6.090
	Fat	1.100	2.030
	Fiber	0.332	0.390
	Carbohydrate	25.516	18.250
	Minerals	0.268	0.133
	Water	45.846	–
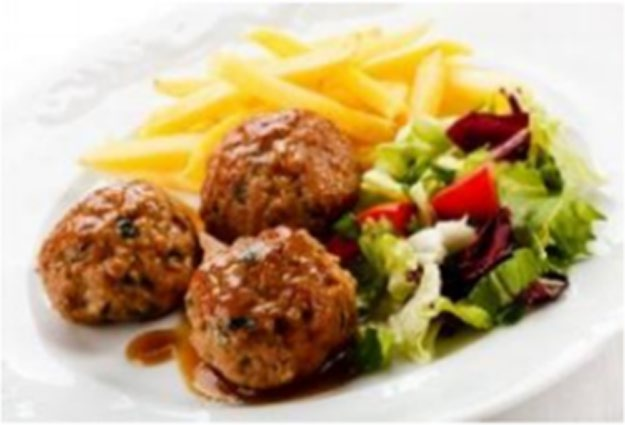	Protein	8.466	4.340
	Fat	6.986	3.380
	Fiber	4.239	1.300
	Carbohydrate	32.207	28.220
	Minerals	0.727	0.292
	Water	36.503	–
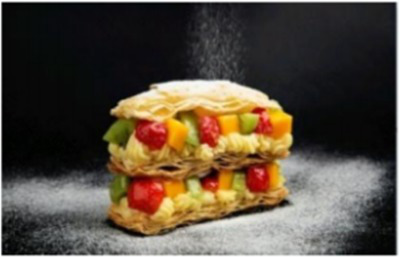	Protein	4.548	3.290
	Fat	20.820	17.080
	Fiber	1.713	1.040
	Carbohydrate	30.351	19.960
	Minerals	0.335	0.273
	Water	37.435	–

### 3.2. Performance test

There are a large number of food image recognition methods, and these works are published in different fields such as computer vision, multimedia, medicine, nutrition and health. In order to compare the performance of the proposed model with other food image recognition, Top-1 and Top-5 classification accuracies are used as evaluation metrics. The Top-1 classification accuracy represents the proportion of test images in which the category with the largest predicted probability matches the actual corresponding nutrient composition. The Top-5 classification accuracy represents the proportion of the Top 5 categories with the largest predicted probability in the test image that match the actual corresponding nutrient composition. The specific calculation formula is as follows:


$$TOP_{1\;Acc}=1-\frac{\left|X_{p}-X_{r}\right|}{X_{r}}$$

$$TOP_{5\;Acc}=\overset{5}{\underset{i=1}{\sum }}\left(1-\frac{\left|X_{ip}-X_{ir}\right|}{X_{ir}}\right)*\frac{1}{5}$$

In addition, the performance evaluation includes two Settings: 1-crop and 10-crop, which denote 1 and 10 cropping for data augmentation, respectively (Table 2).

**Table 2:** Performance comparison of DelicacyNet with other models.

Methods	Settings	Recipe 1 M+	Food2K			Top-1 Acc.	Top-5 Acc.	Top-1 Acc.	Top-5 Acc
GoogLeNet (36)	1-crop	49.13	52.91%	57.15	32.91%
VGG-16 (37)	1-crop	61.15	57.15%	77.42	49.04%
SENet154 (38)	1-crop	75.33	70.17%	82.93	82.13%
SGLANet (39)	1-crop	76.24	74.56%	83.31	81.37%
ReXNet (40)	1-crop	75.14	77.12%	84.52	82.95%
SGTN (41)	1-crop	75.56	76.15%	74.71	78.62%
PAR-Net (42)	10-crop	75.91	72.91%	83.30	81.48%
LocalViT (43)	1-crop	79.99	79.65%	85.87	83.50%
MPViT (44)	1-crop	80.73	79.01%	85.42	84.11%
DelicacyNet(ours)	1-crop	81.26	79.33%	87.53	84.53%

We trained the model on the food2K dataset, and used Recipe1M+ and Food2K, respectively, to test and evaluate the model, and the amount of data tested was about 50,000. The results showed DelicacyNet had good performance. It’s worth mentioning that if a model does not predict the presence of some nutrients, then it will have a score of 0, and if this happens too often, the final prediction accuracy will be very low.

### 3.3. Ablation experiment

We performed ablation experiments to verify the effectiveness of using the EFE module. Table 3 shows the model’s predictions for food fats, carbohydrates, and proteins when the EFE module is added and removed. The stability of the model increased with the addition of the EFE module. And Supplementary Figure S2 shows the prediction accuracy of the model with EFE module is more stable during training. In the several groups of images selected in Table 3, since the food in the image does not change, the prediction of the model should be as stable as possible. That is, the smaller the variance of the prediction for the two pictures is, the better the anti-environmental interference ability of the model is.

**Table 3:** The degree of stability of the model predictions for different backgrounds without or with the EFE module.

		Without EFE module		With EFE module	
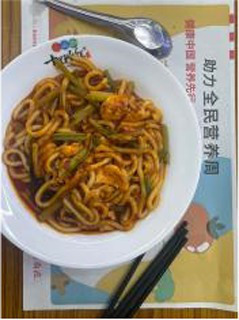 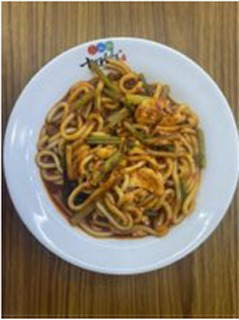	Fat	12.90%	6.68%	5.08%	4.88%
	Pro.	1.99%	8.21%	8.61%	8.42%
	Car.	50.52%	74.69%	77.92%	80.09%
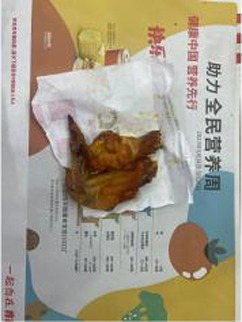 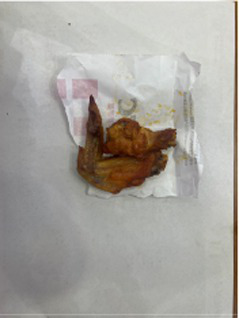	Fat	13.32%	11.68%	12.74%	11.92%
	Pro.	6.15%	21.30%	20.45%	18.23%
	Car.	40.77%	20.80%	30.66%	29.37%
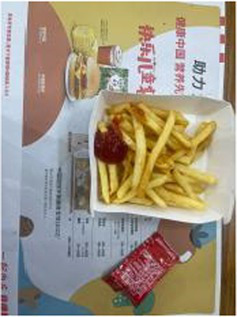 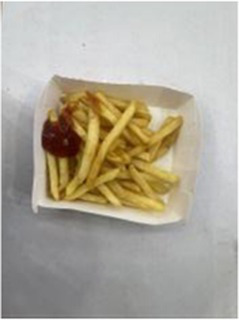	Fat	10.67%	15.42%	12.08%	8.12%
	Pro.	4.18%	8.55%	4.86%	3.37%
	Car.	50.27%	31.80%	36.42%	38.03%

The predicted value in the table is the net content of the ingredient in the food. The larger the predicted gap is for each group of pictures, the more unstable the model prediction is.

By increasing the number of decoding layers, we also compared its effect to prove its effectiveness. Table 4 and Supplementary Figure S3 show the model’s predictions for food fats, carbohydrates, and proteins when different numbers of decode layers are added.

**Table 4:** The model’s predictions for nutrients when different numbers of decode layers are added.

		Fewer decoding layers		More decoding layers	
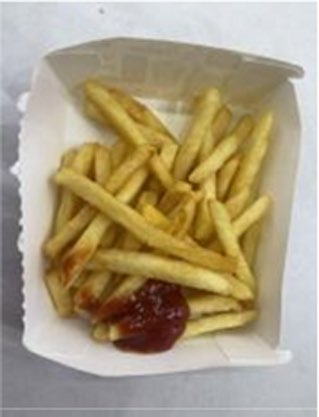 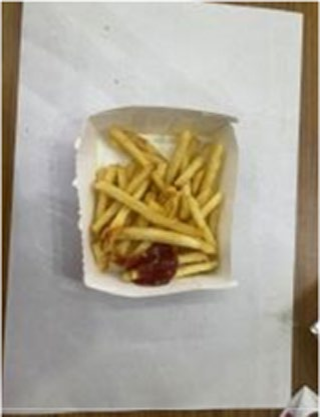	Fat	8.30%	7.76%	8.37%	8.12%
	Pro.	4.16%	3.07%	3.58%	3.37%
	Car.	38.10%	38.55%	38.81%	38.03%
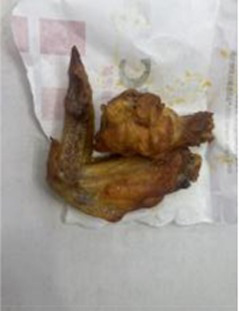 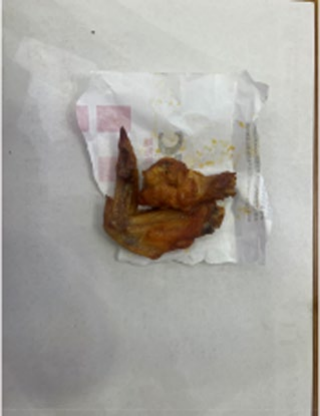	Fat	11.63%	10.89%	11.26%	11.92%
	Pro.	20.44%	19.98%	18.49%	18.23%
	Car.	29.43%	28.63%	29.73%	29.37%
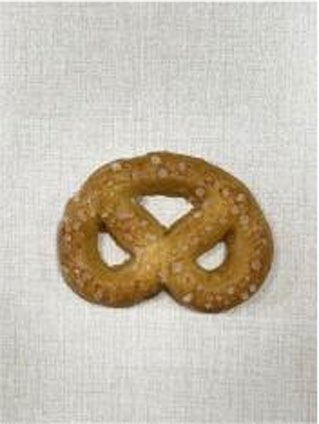 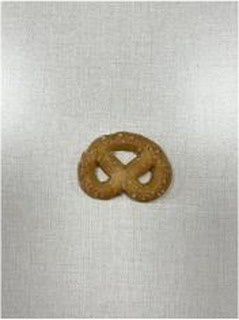	Fat	12.81%	11.98%	12.70%	10.73%
	Pro.	8.35%	7.12%	9.30%	8.03%
	Car.	70.46%	70.89%	70.70%	70.41%

The left side shows how stable the model predictions are for images of different sizes when stacking a smaller number of modules. The right side shows how stable the model predictions are for images of different sizes when a larger number of modules are stacked. The predicted value in the table is the net content of the ingredient in the food. The larger the predicted gap is for each group of pictures, the more unstable the model prediction is.

## 4. Conclusion

We designed an innovative neural network architecture DelicacyNet, which included the following four main modules: environment feature extraction module, encoder, decoder, and semantic output module. After inputting food pictures, we analyzed and obtained the main nutrients contained in the raw materials of the food. After receiving the image, our program first extracted the environmental features, then performed special processing on the features through the encoder, and finally output the obtained features in the form of a text table through the decoder. Our model had high accuracy in the process of predicting food components, and can be applied in practice.

At present, our model can help humans understand the content and proportion of nutrients in their daily intake, so as to selectively decide the nutrition and energy intake in food, and help humans rationally match the required diet. Next step, we hope that our program can expand into new fields, for example, the type and content of raw materials of food can be obtained through the program, and the data of major allergens can be combined to help human prevent diseases.

## Data availability statement

The original contributions presented in the study are included in the article/Supplementary material, further inquiries can be directed to the corresponding author.

## Author contributions

RL: investigation, data curation, and writing-original draft preparation. PJ: methodology, investigation, and writing-original draft preparation. QK: conceptualization, funding acquisition, and writing – review & editing. All authors contributed to the article and approved the submitted version.

## Funding

This study was supported by the National Key R&D Program of China (2019YFD0901705).

## Conflict of interest

The authors declare that the research was conducted in the absence of any commercial or financial relationships that could be construed as a potential conflict of interest.

## Publisher’s note

All claims expressed in this article are solely those of the authors and do not necessarily represent those of their affiliated organizations, or those of the publisher, the editors and the reviewers. Any product that may be evaluated in this article, or claim that may be made by its manufacturer, is not guaranteed or endorsed by the publisher.
