# Supplementary material for: DelicacyNet for nutritional evaluation of recipes
Source: Front Nutr. 2023 Sep 14;10:1247631. doi: 10.3389/fnut.2023.1247631 (PMC10537284; doi:10.3389/fnut.2023.1247631)
Supplement: Supplementary file 1 [file Data_Sheet_1.docx]

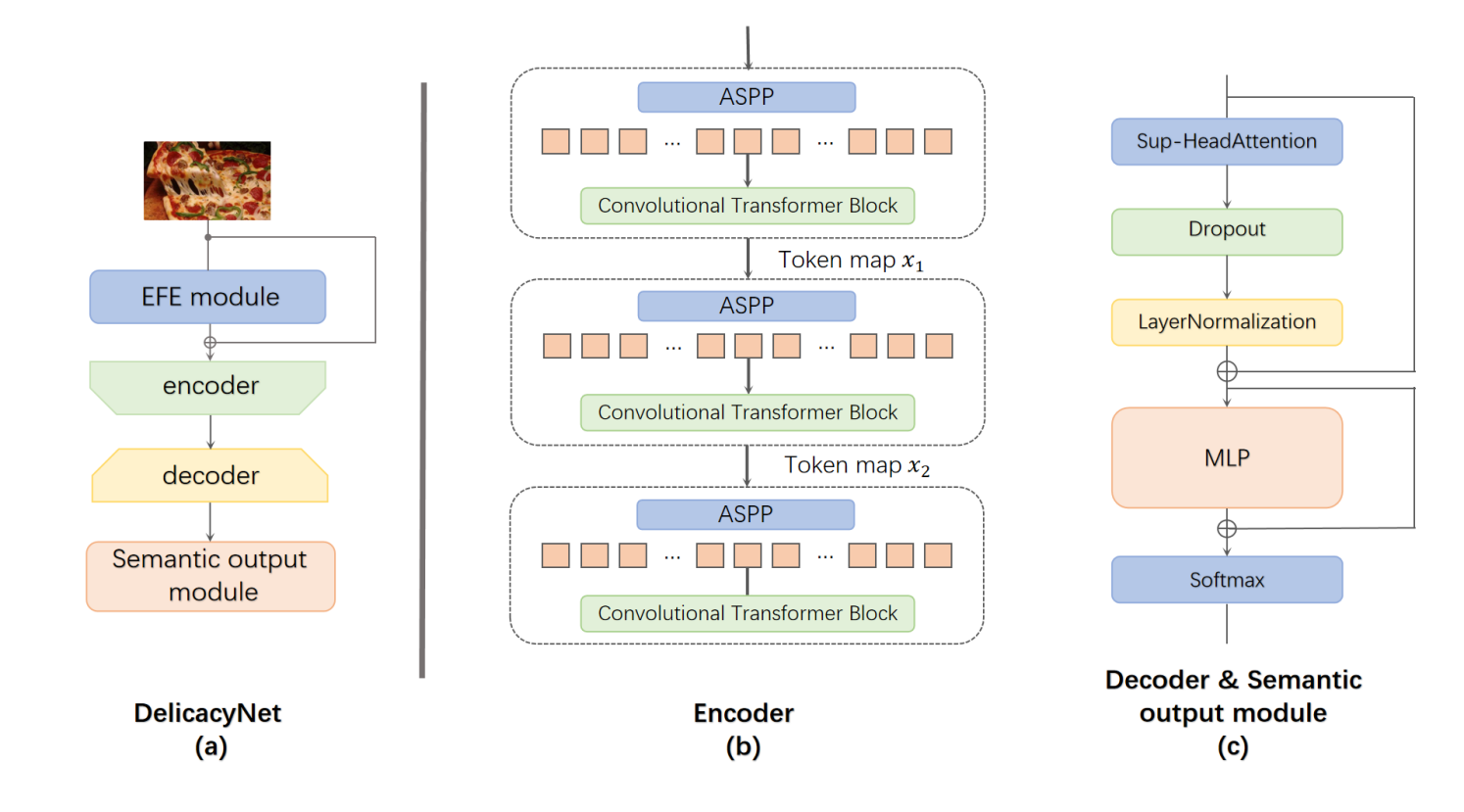


Supplementary Figure S1 Structure of the DelicacyNet (a) consisting encoder (b), decoder, and semantic output module (c).

(a)


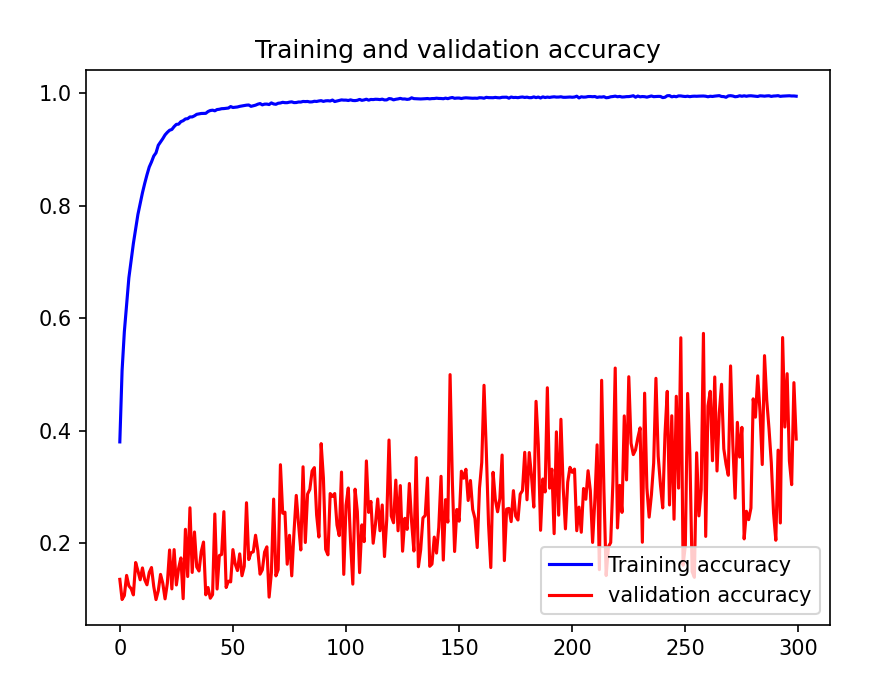

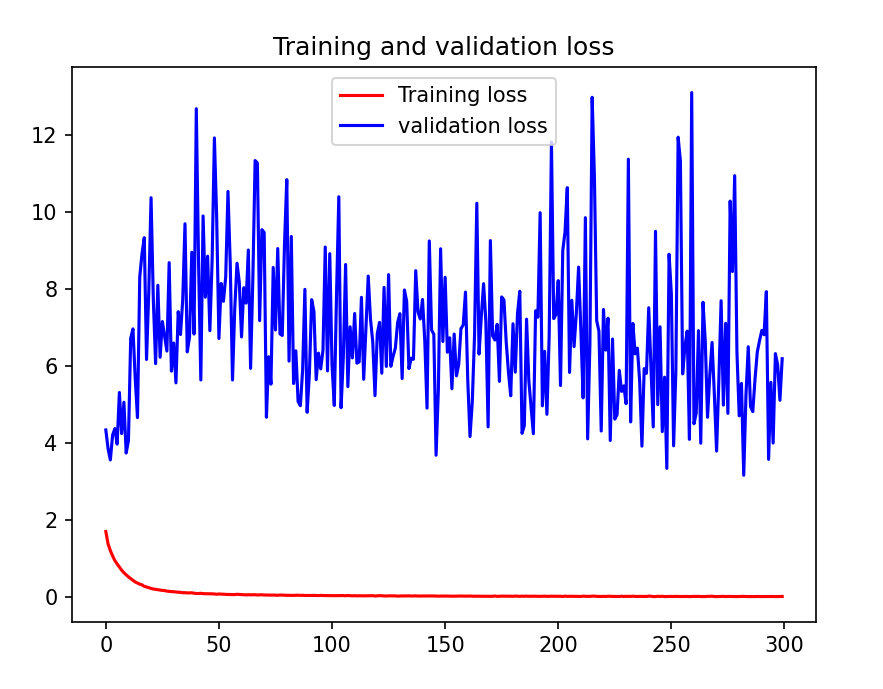


(b)


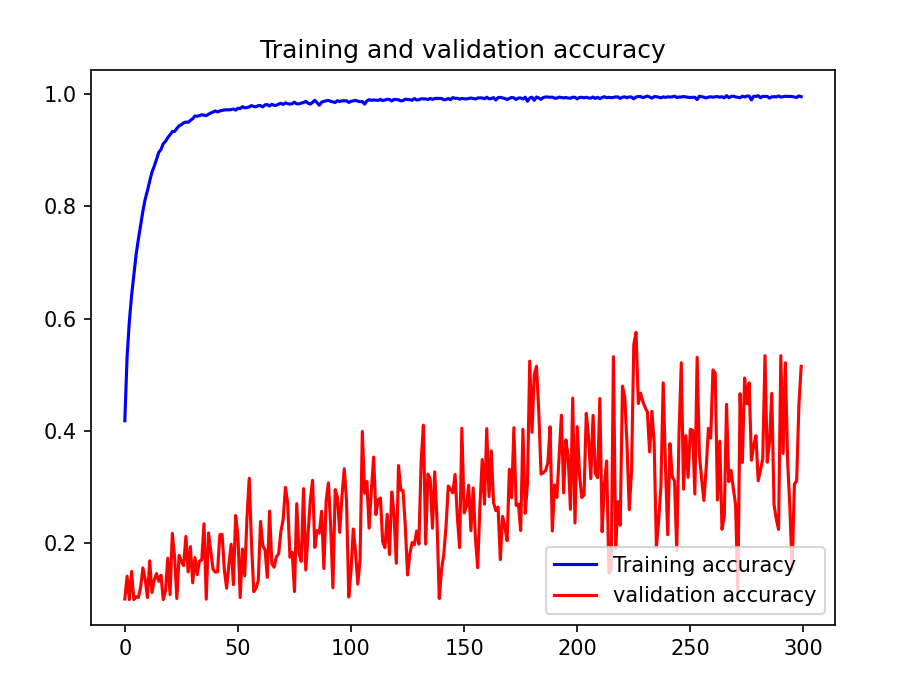

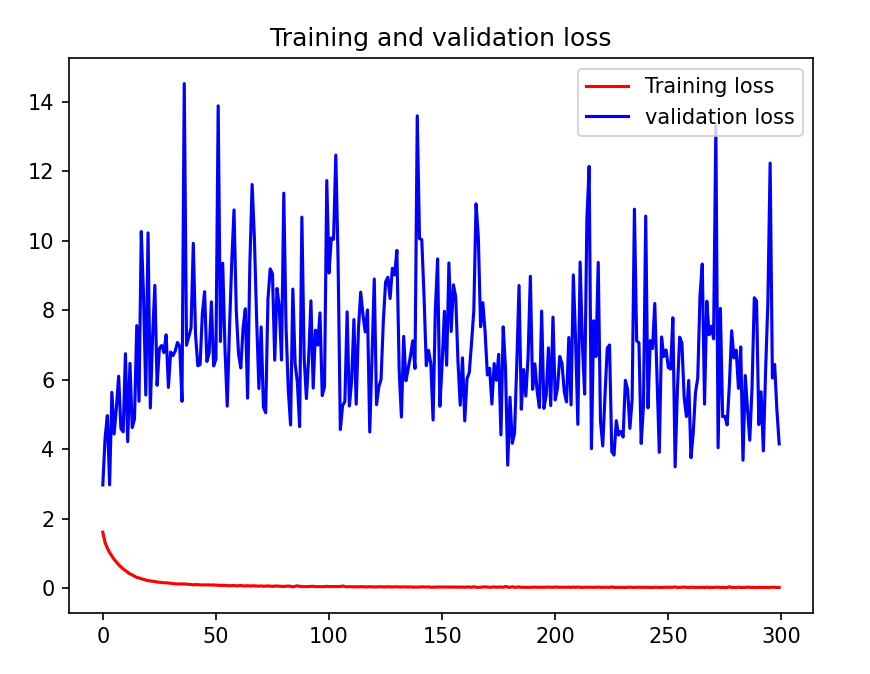


Supplementary Figure S2 The prediction accuracy of the model without EFE module (a) and with EFE module (b). Data shown in the first 300 iterations.

(a)


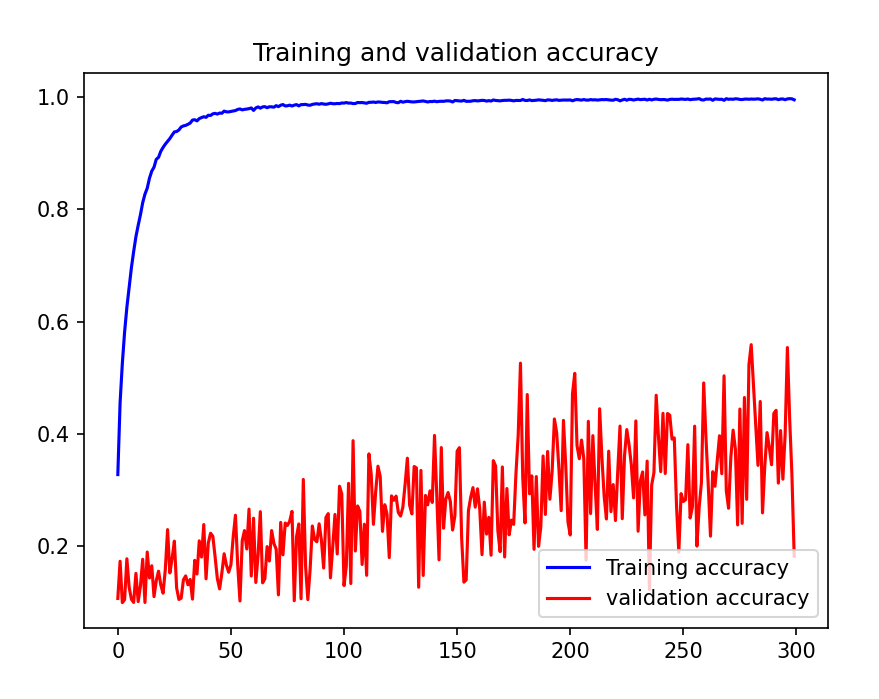

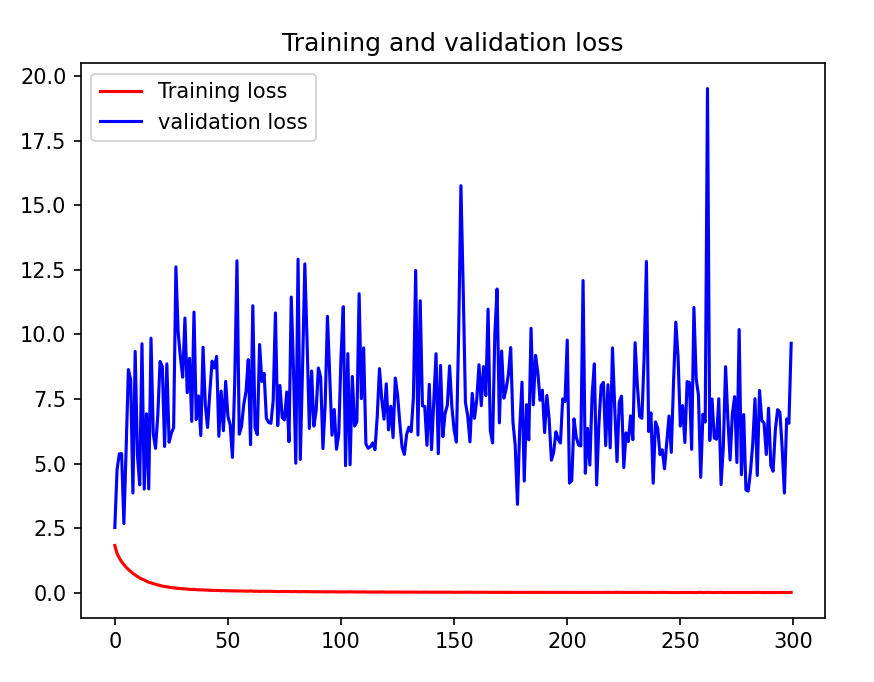


(b)


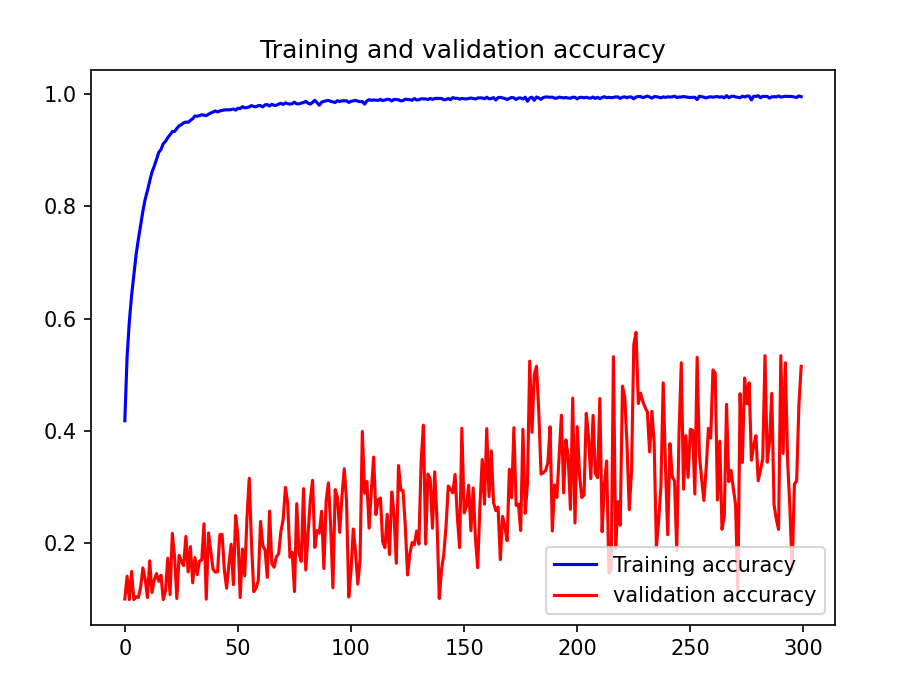

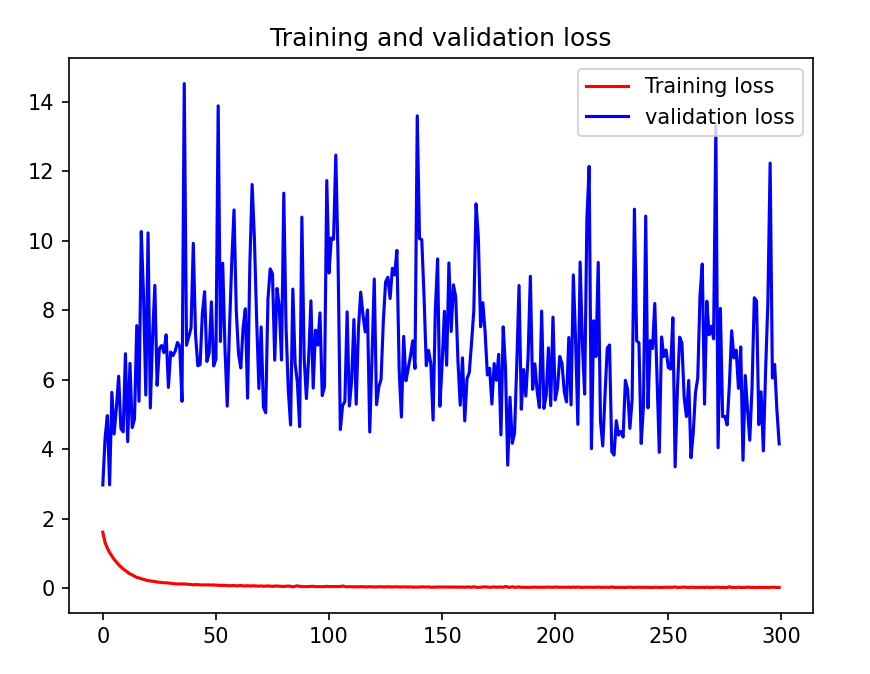


Supplementary Figure S3 The prediction accuracy of the model when stacked fewer decoding layers (a) and stacked with more decoding layers (b). Data shown in the first 300 iterations.
